# Rethinking glioma treatment strategy

**DOI:** 10.18632/oncotarget.2619

**Published:** 2014-10-22

**Authors:** Tatsuya Ozawa, Eric C. Holland

**Affiliations:** Division of Human Biology and Solid Tumor Translational Research (STTR), Fred Hutchinson Cancer Research Center, Department of Neurosurgery and Alvord Brain Tumor Center University of Washington; Division of Human Biology and Solid Tumor Translational Research (STTR), Fred Hutchinson Cancer Research Center, Department of Neurosurgery and Alvord Brain Tumor Center University of Washington

Over the past decade, deep molecular analysis has revealed that many cancers, including glioblastomas (GBMs), can be subdivided in several subtypes according to genetic and/or transcriptomal similarity. These subtype classifications have given significant insight into tumor biology and provided encouragement for targeted therapeutic strategies. In fact, molecular target therapies have conferred therapeutic benefit in certain cancer types as demonstrated by Imatinib treatment to BCR-ABL mutant CML or KIT mutant GIST (Kantarjian et al., 2002; Miettinen and Lasota, 2006). Given that there are druggable canonic mutations such as EGFR and PDGFRA enriched in specific subtypes, one would think that these mutation(s) should be possible candidates for subtype-specific molecular target therapy. However, molecular target therapies against these mutations in GBMs have not been successful, even in GBMs with those genetic alterations (van den Bent et al., 2009; Wen et al., 2006). The disappointing result in GBM suggests that we may need to reexamine our assumptions regarding how the molecular subtypes would be expected to correlate with patient treatment response.

Another hope for dividing these tumors into groups with predictable therapeutic responses is by molecular subtype. GBMs can be generally segregated into 3 or 4 subtypes according to Verhaak’s and Phillips’s gene expression signature (Phillips et al., 2006; Verhaak et al., 2010). The credibility of these gene expression signature based groups is that they enrich for some of these druggable mutations. However, they are partly overlapping, the border between them is vague, and in some cases regional subtype mosaicism is also observed in same tumor (Sottoriva et al., 2013). Which subtype we should target for patient at the border between subtypes or with multiple subtypes? In fact, recent cumulative evidences suggest that the distinction between molecular GBM subtypes is not necessarily robust, rather variable throughout entire process of tumor formation. It was observed that PN-GBM phenotype can spontaneously convert to MES-GBM phenotype over time in human patients (Phillips et al., 2006). Further single cell RNA sequencing clarified that GBMs were heterogeneous mixtures of individual cells with different GBM types at single cell level, and cells presenting a PN gene expression signature were included in all tumors analyzed regardless of the GBM subtype determined with the tumors (Patel et al., 2014). Our phylogenetic analysis reveals that all 4 of the non-GCIMP GBM subtypes evolve from a precursor GBM that likely has a PN phenotype. Mathematical and experimental mouse modeling demonstrated that GBMs were initiated as the ancestor subtype: PN-GBM and branched to other subtypes upon an acquisition of subtype specific mutation(s) at a later stage of the tumor formation (Ozawa et al., 2014). Thus GBM subtypes based on expression signature seem to represent a dominant phenotype of heterogeneous cell population in the tumor and to be temporary phenotype in a dynamic state throughout tumor formation. If this is actually the case, the GBM subtypes are unlikely indicators for molecular target therapy.

Focal genomic alterations and recurrent broad gains and losses across the GBM genome are well known, but whole chromosome aneuploidy represented by chr7 gain and chr10 loss are actually the most prominent features in the non-GCIMP GBM genome, found in approximately 80% of all subtypes (Ozawa et al., 2014). However whether aneuploidy is a cause or a consequence of tumorigenesis is still controversial. The presence of extra chromosomes results in the increased-expression of many genes on the chromosome, in some cases oncogenic ones, and aneuploidy indeed has a tumor-promoting effect under certain contexts (Weaver and Cleveland, 2006; Williams et al., 2008). On the other hand, despite its high prevalence in human cancers, experimental models indicate that aneuploidy itself can be deleterious for normal cells and in some circumstances exhibit anti-tumor effect rather than enhances tumorigenesis (Weaver and Cleveland, 2008). In the case of GBM, ch7 gain and ch10 loss are calculated by mathematical modeling to be the first event in the population of cells that make up the tumor at the time of diagnosis. The gene on ch7 that correlates copy number and expression and patient survival best is PDGFA, which is also sufficient to induce gliomas in mice. (Ozawa et al., 2014)

There are significant implications for therapy in these observations. Although PDGFA is a main driver for gain of ch7, there are many other genes on ch7 and ch10 that contribute to these tumors right from the beginning. Furthermore the druggable targets associated with the molecular subtypes, such as amplification of PDGFRA and mutation of EGFR, are late events in the evolution of the disease, occurring in tumors that were already lethal at the time they occurred and are represented heterogeneously in these tumors at the time of diagnosis. It is not surprising that targeting these mutations has not worked in GBM. Rather than narrowly treating specific mutations and subtypes, we should be looking for therapies that address the biology of gliomas as a whole. We should target the things that unify this disease rather than those that appear to subdivide it.

